# Proton beam therapy for clival chordoma: Optimising rare cancer treatments in Australia

**DOI:** 10.1002/jmrs.774

**Published:** 2024-03-19

**Authors:** Ashwathy Mathew, Peter Gorayski, Nicholas Candy, Frank Saran, Hien Le

**Affiliations:** ^1^ Department of Allied Health and Human Performance University of South Australia Adelaide South Australia Australia; ^2^ Australian Bragg Centre for Proton Therapy and Research South Australian Health and Medical Research Institute Adelaide South Australia Australia; ^3^ Department of Radiation Oncology Royal Adelaide Hospital Adelaide South Australia Australia; ^4^ Department of Surgery The University of Adelaide Adelaide South Australia Australia; ^5^ Department of Neurosurgery Royal Adelaide Hospital Adelaide South Australia Australia; ^6^ Department of Surgery‐Otolaryngology, Head & Neck Surgery University of Adelaide Adelaide South Australia Australia

**Keywords:** cancer, Chordoma, proton therapy, radiation therapy, rare cancers, sarcoma

## Abstract

With the anticipated launch of the Australian Bragg Centre for Proton Therapy and Research (ABCPTR) in Adelaide, Australia, proton therapy will become a significant addition to existing cancer treatment options for Australians. The anticipated benefits will be particularly evident in rare cancers such as clival chordomas, a challenging tumour entity due to the anatomical relationship with critical structures, and proven radio‐resistance to conventional radiation therapy. The article synthesises key findings from major studies and evaluates the current evidence supporting various management strategies for clival chordomas. It also considers the influence of institutional volume and multidisciplinary team management on patient outcomes and outlines how high‐quality care can be effectively delivered within the Australian healthcare system, emphasising the potential impact of proton therapy on the treatment paradigm of clival chordomas in Australia.

## Graphical abstract

The article discusses the anticipated launch of the Australian Bragg Centre for Proton Therapy and Research (ABCPTR) in Adelaide, highlighting the potential benefits of proton therapy for rare cancers like clival chordomas. It synthesises key findings from major studies, evaluates management strategies for clival chordomas, considers the role of institutional volume and multidisciplinary teams in patient outcomes, and explores how high‐quality care can be delivered in the Australian healthcare system, emphasising the potential impact of proton therapy on clival chordoma treatment in Australia.

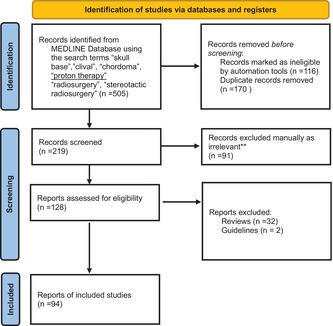

## Introduction

Chordomas are rare, malignant, locally aggressive neoplasms that typically originate in the axial skeleton, embryologically arising from the remnants of the notochord. The estimated number of Australian adult patients with skull base chordomas in 2025 is expected to be around 31 per year, making it a rare tumour within the Australian context.^1^, ^2^ Skull base chordomas account for 26–32% of all chordomas.^3^, ^4^ Of these, 49% arise in the clivus, 32% in the sphenoclival region, 9% in the petroclival region, 6% in the cervical spine, and 4% in other areas. In children and young adults up to the age of 20, chordomas are more frequently located at the skull base (70%) compared to older patients (21%).^1^, ^3^ Unless detected incidentally, patients frequently present at an advanced stage of their disease, characterised by sizable tumours. The primary surgical objective of achieving a complete excision becomes exceptionally challenging due to the frequent infiltration of the brainstem, cranial nerves, and major blood vessels by the tumour.^3^ Adding to the challenge of achieving optimal local control and overall survival rates is the inherent ‘radio‐resistance' of these tumours, as evidenced by a low a/b ratio of approximately 2.45,^5^ necessitating the administration of elevated radiation doses for effective tumour control. Consequently, the primary recurrence pattern observed in skull base chordomas is local relapse, either as the sole site of recurrence or as a component of failure, accounting for the leading cause of death in approximately 95% of patients.^6^

The standard treatment approach for this locally invasive tumour has historically revolved around either pursuing gross total resection or achieving the maximum safe surgical removal, followed by adjuvant radiotherapy to a dose of 55–80 Gy.^7^, ^8^ Over the past few decades, there has been a gradual improvement in outcomes for clival chordoma patients, owing to advancements in surgical techniques and the evolution of radiation therapy modalities and delivery methods. Previous literature reported a mean survival of 5.2 years,^9^ compared with contemporary case series reporting mean overall survival and mean progression‐free survival at 7 years of 71% and 31%, respectively.^10^ Due to the low incidence of this malignancy, high‐quality evidence from randomised clinical trials is lacking. Therefore, much of the current evidence is based on large case series from higher volume centres of excellence.^11^

It is generally accepted that cumulative radiation doses over 70 Gy are needed to achieve optimal local control.^12^ Such high radiation doses in the vicinity of many critical organs at risk carry a higher risk of treatment‐related toxicities. These include brainstem necrosis, radiation‐induced optic neuropathy, hypopituitarism, multiple cranial nerve palsies, and temporal lobe necrosis.^13^, ^14^ Radiation oncologists are continually challenged to balance the delivery of high doses to the tumour with the imperative to keep the risk of radiation‐induced toxicities within clinically acceptable limits. Where available, proton therapy has been internationally established as an integral part of the treatment of clival chordomas, owing to its distinct physical and dosimetric properties that enable the precise delivery of high‐dose radiation to the tumour, while minimising exposure to the surrounding normal tissues.^11^, ^15^, ^16^ This targeted approach translates into enhanced local control and an increased likelihood of cure in skull base chordomas.^17^

Australia is poised to introduce its first proton therapy unit at the Australian Bragg Centre for Proton Therapy and Research (ABCPTR) in Adelaide, South Australia.^18^ This milestone signifies a pivotal advancement in the care of patients diagnosed with clival chordomas and other rare cancers within the Australasian healthcare landscape. It promises enhanced alignment with international best practices and represents a major leap forward from the existing Medical Treatment Overseas Program (MTOP), offering the potential for domestic treatment for these patients.^19^ It also offers a unique opportunity to generate long‐term, high‐quality data regarding the treatment and outcomes of chordoma at the ABCPTR, which consequently will become a high‐volume quaternary referral centre for this rare tumour entity. In this context, the existence of an experienced skull base surgery programme and the integration of the Australian Particle Therapy Clinical Quality Registry (ASPIRE) for prospective collection of long‐term outcomes from patients treated with proton therapy is both timely and aspirational.^20^

This article critically evaluates the current evidence and developments in proton therapy, with a specific emphasis on its application in managing clival chordoma within the context of Australia. The overarching theme of this narrative review is the utilisation of radiation therapy in the treatment of clival chordoma, with a particular focus on proton therapy and its associated evidence base.

### Literature review

**Figure 1:**
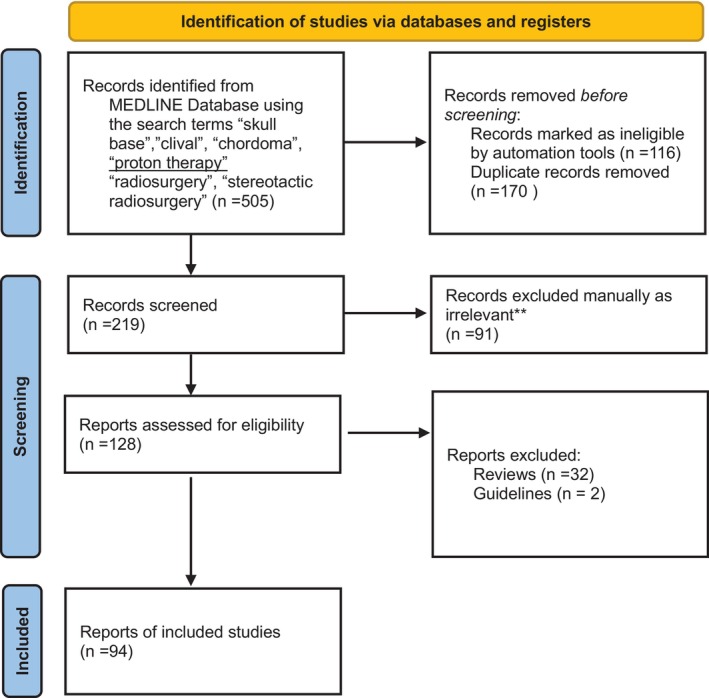
PRISMA flow diagram of Screening Process for studies included in review. Modified from: Page MJ, McKenzie JE, Bossuyt PM, Boutron I, Hoffmann TC, Mulrow CD, et al. The PRISMA 2020 statement: an updated guideline for reporting systematic reviews. BMJ 2021;372:n71. doi: 10.1136/bmj.n71.

We conducted a comprehensive search of the MEDLINE database, employing relevant MESH terms including ‘skull base’, ‘clival’, ‘chordoma’, ‘radiosurgery’, and ‘proton therapy’, as well as several related variations. The search results underwent a screening process to eliminate duplicates and filter for articles in English, specifically focussing on research involving human subjects. Details of the screening process are illustrated in Figure 1. Out of a total of 505 search results, 219 publications were screened for relevance to the overarching theme of this narrative review. Our focus centred on original research articles that provided comprehensive institutional experiences in the primary treatment of clival chordomas, specifically those involving moderately large patient cohorts. We included studies exploring various radiation modalities, including photon, proton, and/or carbon ion therapies. Excluded from our review were studies directly comparing surgical approaches and those addressing the treatment of recurrent disease. Of the 128 reports retrieved, 94 articles were reviewed in detail, and the most relevant were included in Table 1 for proton therapy and Table 2 for photon‐based RT. The evidence for surgery in this cohort was examined as it relates to the primary topic of radiation therapy.

**Table 1:** Selected series using proton therapy for skull base chordomas.

Authors (year of publication)	Type of study (Institution)	*N* (skull base chordomas only)	Local control rate	Overall survival rate	Toxicities	Comments
*Retrospective institutional series*						
Ares et al.^40^ (2009)	Retrospective case series (PSI)	41	5 yr = 81%	5 yr = 62%	Late toxicity ≥Gr 3 in 4 pts	Mean prescribed dose = 73.5 GyE
Deraniyagala R et al.^47^ (2014)	Retrospective case series (UFHPTI)	33	2 yr – 86%	2 yr – 92%	Late toxicity ≥Gr 3 – nil	Mean prescribed dose = 77.4–79.4 GyE
Weber DC et al.^16^ (2016)	Retrospective case series (PSI)	151	5 yr = 75.8% 7 yr = 70.9%	5 yr = 86.4% 7 yr = 73%	Late Gr 3–4 toxicity – 8%. No Gr 5 toxicity. No secondary malignancy	Mean dose = 72.5 GyE+/− 2.2 GyE
McDonald et al.^37^ (2016)	Retrospective case series (IUHPTC)	39	5 yr = 69.6% (95% CI = 65.3–97.5%)	5 yr = 81.4% (95% CI = 65.3–97.5%)	Acute ≥Gr 3 toxicity – 1 patient; Late toxicity ≥Gr 3–3 patients (sensorineural hearing loss and radiation necrosis)	Median dose = 77.4 GyE. Local failures had significantly larger GTV and lower D1cc and prescribed and median doses to GTV. LC was associated with smaller residual tumour and more complete high‐dose coverage of the residual tumour. Authors proposed a minimum dose of 74.5 GyE to the D1cc of GTV as planning objective
Holtzman et al.^48^ (2021)	Retrospective case series (UFHPTI)	112	Actuarial 5‐yr LC = 74%	Actuarial 5‐yr OS = 78%	Acute ≥Gr 3 toxicity – nil; Late toxicity – 17% hypopituitarism; <3% ≥ Gr 3 vision loss; 5% Gr 3 temporal lobe injury; 2% Gr 5 potentially radiation attributed events	95% definitive/adjuvant treatments; 93% pure proton treatment; median dose 73.8 Gy RBE (range 69.6–75.6 Gy RBE)
Youn SH et al.^15^ (2018)	Retrospective case series (NCC, Korea)	58 (37 = skull base)	5‐yr LPFS = 97% for skull base	5‐yr OS = 88.3% for whole cohort	Gr 3 late toxicity = 3 patients; no ≥Gr 4	Median dose = 69.6 CGE (range 64.8–79.2 CGE); lower LPFS for skull base‐cervical tumours than non‐cervical tumours
Iannalfi et al.^49^ (2020)	Retrospective case series (CNAO, Italy)	135 skull base chordomas (70 PT or 65 CIRT)	5 yr = 84% with PT, 71% with CIRT	5 yr = 83% for PT pts	Gd 3–4 toxicity = 12%	CIRT mostly for recurrent cases. Median dose with proton therapy 74 GyE; median GTV 3.5 cc(0–99.3)
Dial BL et al.^17^ (2020)	Retrospective database analysis (NCDB)	567 (Proton therapy patients = 189)	NR	5 yr = 84% (95% CI 80–87%)	NR	Patients treated with high‐dose RT >65 Gy and advanced techniques (proton therapy, IMRT, or SRS) had better OS compared to conventional EBRT <65 Gy). Proton beam radiotherapy had improved survival compared to EBRT (non‐IMRT/SRS)
*Meta‐analysis*						
Di Maio et al.^50^ (2011)	Meta‐analysis	807 cranial base chordomas who underwent surgery plus adjuvant radiation	5‐yr PFS = 50.8% 5‐yr PFS weighted for proton therapy = 57% ± 4.8%	5‐yr OS = 78.4% 5‐yr OS weighted for proton therapy alone = 76.6% ± 4.5%	NR	No OS difference between radiation techniques. 5‐yr PFS lower in patients receiving GK‐SRS compared with Carbon ion (*P* = 0.042)

CI, confidence interval; CIRT, carbon ion radiotherapy; CNAO, Centro Nazionale di Adroterapia Oncologica, Italy; D1cc, minimum dose received by the most irradiated 1 cc of the volume; EBRT, external beam radiotherapy; GK, Gamma Knife; GTV, gross tumour volume; GyE, Grey equivalent; IMRT, Intensity‐modulated radiation therapy; IUHPTC, Indiana University Health Proton Therapy Centre, Indiana, USA; LC, local control; LPFS, local progression‐free survival; NCC, National Cancer Centre, Korea; NCDB, National Cancer DataBase; NCDB, National Cancer Database, USA; NR, not reported; OS, overall survival; PFS, progression‐free survival; PSI, Paul Scherrer Institute, Switzerland; PT, proton therapy; SRS, stereotactic radiosurgery; UFHPTI, University of Florida Health Proton Therapy Institute; Yr, year.

**Table 2:** Selected series using photon‐based techniques for skull base/clival chordoma.

Author (Year of Publication)	Type of study (Institution)	N (skull base chordomas only)	Local control rate	Overall survival rate	Toxicities	Comments	
*Retrospective institutional series*							
Kano et al.^29^ (2011); report of North American Gamma Knife consortium	Retrospective pooled multi‐institutional study	71	5‐yr actuarial 66%	5‐yr actuarial 80%	Neurological symptoms deterioration – 4 patients, both tumour progression and acute radiation effects – 2 patients; 9% Gr2‐3 toxicity; no Gr 4 toxicity	Both primary and recurrent (28%) tumours were treated with GK, median margin dose = 15 Gy	
Hauptman JS et al.^30^ (2012)	Retrospective case series (UCLA)	15 skull base tumours (13 chordomas)	9/15 cases showed local control (stable or partial response)	NR – all patients alive	Endocrinopathy (1), cranial neuropathy (2), visual deficits (1), transient medial temporal lobe radiation changes (1)	SRT or SRS on linac, if optic apparatus is included in 80% isodose, advisable to fractionate. Pituitary stalk <30 Gy to minimise endocrine dysfunction. Brainstem <60 Gy fractionated dose	
Bugoci et al.^27^ (2013)	Retrospective case series	12	2‐yr PFS = 46.9%, 5‐yr PFS = 37.5%	5‐yr OS = 76.4%	Nil	Median conventional RT dose 66.6 Gy. Adjuvant or salvage RT (Median time from surgery to RT was 3.6 m so mostly adjuvant); dynamic conformal arcs and IMRT boost till 2006 then intensity‐modulated FSRT	
Ahmed R et al.^33^ (2015)	Retrospective case series	49 chordomas (30 skull base)	5‐yr PFS =70% in clival chordomas	5‐yr OS = 73% 10‐yr OS = 44% – in clival chordoma	NR	Adjuvant high‐dose stereotactic fractionated RT (HS‐FSRT) significantly improved 5‐yr PFS in craniocervical chordoma (70%) compared to standard dose RT (20%). 10‐yr OS was same for both groups (40–45%). Hyperfractionated BID treatment to 60 Gy followed by boost to 21 Gy (1.5 Gy BID)	
Hafez et al.^34^ (2019)	Retrospective case series	12 post‐operative residual clival chordoma	5‐yr actuarial LC = 25% (at mean FU of 45 months, 66.7% failed)	41.7% died. Mean follow‐up = 26.5 months	Nil	Failed patients mean tumour volume was 9.2 cc with mean peripheral dose of 13.5 Gy compared to controlled tumours whose mean tumour volume was 2.7 cc with mean peripheral prescription of 16 Gy	
Shinya et al. (2022)^35^	Retrospective case series	47	5‐yr LC = 63%; High‐dose SRS cohort (≥20 Gy marginal dose) 5‐yr LC = 73%	5‐yr OS 83% for entire cohort; 100% for ‘extended‐field SRS’ group	1 patient had Gr 3 motor neuropathy (undergone 3 SRS for persistent tumour recurrence)	Initially ‘localised field SRS’ to include residual/recurrent tumours only from 1990 to 2015; After 2015 ‘extended‐field SRS’ (residual tumour + pre‐operative tumour location +2 mm margins) treated to marginal doses 18–20 Gy; marginal field doses ≥20 Gy had better local control. Extended‐field SRS improved remote control rates (outside 20% isodose line), not local control rate	
Pikis et al. (2022)^31^; update of North American Gamma Knife consortium study – Kano et al. (2011)	Retrospective case series	93	5‐yr PFS = 54.7% 10‐yr PFS = 34.7%	5‐yr OS = 83% 10‐yr OS = 70%	3.2% adverse radiation effects; 16.1% new neurological deficits; 4.3% new pituitary endocrine axis loss	82.8% were adjuvant treatments; mean tumour volume 8 cc; mean margin dose 17 Gy (SD 3.6); max dose >29 Gy associated with better tumour LC (*P* = 0.02)	
*Systematic review*							
Bin‐Alamer Othman et al.^43^ (2022)	Systematic review – statistical analysis unclear	130 patients of skull base chordomas treated with SRS (15 articles)	5‐yr LPFS of entire cohort = 9%; median PFS of primary RT cohort – 24 months(IQR – 15–34 m)	5‐yr OS of entire cohort = 82%; median OS of primary RT cohort – 293 months (IQR – 137.4 m‐not reached)	SRS‐related side effects (*n* = 10)‐cranial nerve neuropathy (2), diplopia and gait disturbance (1), dizziness (1), neurological deterioration (1), dysphagia, facial numbness, dysarthria and decreased visual acuity (1), unspecified endocrinopathy, unspecified cranial nerve neuropathy, hemiparesis (1), diplopia (1), visual field deficits and hearing loss (1), headache (1)	36 pts (27.7%) had prior irradiation, possibly recurrent tumours. 72.3% primary RT, rest for recurrent/reirradiation	

BID, twice daily fractionation; CI, confidence interval; CIRT, carbon ion radiotherapy; CN, cranial nerve; D1cc, minimum dose received by the most irradiated 1 cc of the volume; EBRT, external beam radiotherapy; GK, Gamma Knife; GTV, gross tumour volume; Gy, Grey; GyE, Grey equivalent; IMRT, Intensity‐modulated radiation therapy; LC, local control; NCDB, National Cancer DataBase; NR, not reported; OS, overall survival; PFS, progression‐free survival; PT, proton therapy; SD, standard deviation; SRS, stereotactic radiosurgery; SRT, stereotactic radiation therapy; UCLA, University of California Los Angeles.

### Review of evidence regarding surgical resection

Any optimal surgical plan requires considerations of three key factors: patient factors (age, co‐morbidities, previous treatment), anatomical factors (dural invasion, major vessel encasement, location, and size), and surgeon factors (experience with complex skull base approaches).^7^ Ultimately, the surgical treatment's overall goal is safe maximal resection while minimising surgical morbidity and preserving quality of life. Rarely is en bloc resection (R0) achievable for any skull base chordoma due to the dense and eloquent nature of adjacent anatomy.^21^ Therefore, the role of adjuvant radiotherapy in treating tumour that cannot be safely resected and/or ensuring eradication of microscopic (R1) or macroscopic (R2) residual disease is imperative.

There are no randomised controlled trials comparing different surgical approaches, with evidence being limited to institutional case series.^22^ The management strategy for approaching these tumours has shifted from exclusively open microsurgical approaches to implementing a 360° approach to each tumour following the development of extended endoscopic approaches.^23^ These newer techniques have improved the degree of resection.^21^ The degree of resection at the index operation is well established as an important predictor of outcome, with complete resection having the best outcomes.^17^ Passeri et al. (from Lariboisière Hospital) and Wang et al. are two of the largest contemporary single‐institution case series published on skull base chordoma.^10^, ^21^ These centres reported 5‐year overall survival and progression‐free survival rates of 75.1% to 76% and 52.1% to 45%, respectively. Even at the Lariboisière Hospital, which is a globally recognised centre with special expertise in the management of skull base chordoma, a gross total resection was only achieved in 43.8% of patients, with a near total resection (>90% resection) being achieved in a further 34.3%. This series highlights the value of adjuvant radiotherapy, as their series demonstrated a significantly lower progression‐free survival in patients who did not receive adjuvant radiotherapy compared with patients who received radiotherapy.^21^

### Review of evidence regarding use of radiotherapy

Initial research demonstrated that conventional fractionated doses in a dose range of 25–60 Gy were insufficient to achieve a high probability of long‐term control.^24^ These patients had a median time to progression of 35 months and overall medial survival of 62 months. With the advent of radiosurgery, it was recognised that small skull base chordomas which had sufficient space between tumour and critical organs such as brainstem could be well controlled with single‐fraction stereotactic radiosurgery (SRS).^25^ Early series reported 2‐year local control rates of up to 82%.^5^, ^26^, ^27^, ^28^, ^29^, ^30^ However, more recent series consistently show 5‐year local control rates ranging between 45% and 73% (Table 2).^31^, ^32^, ^33^, ^34^, ^35^ Of note, Shinya et al.^35^ report 5‐year local control rates of 63–73% only when treating larger volumes including the pre‐operative tumour volume with margins to margin doses of 18–20 Gy. Irradiating only the gross tumour residual/recurrences led to unacceptably high local recurrence rates, even when irradiating to high marginal doses, as recurrences occurred in the surrounding surgical cavity.

Proton therapy was used in specialised centres for skull base chordomas to attempt dose escalation in this challenging anatomical site, initially with passive‐scattered proton therapy, and later with pencil‐beam scanning proton therapy techniques. The ability to provide durable control of the primary tumour is dependent on the amount of residual tumour post‐resection and the ability to deliver very high radiation dose to the residual disease, as underdosing even a small part of the target volume has been associated with a reduced probability of local control.^12^, ^36^, ^37^ The importance of delivering high doses to the tumour to ensure local control is seen in the study by Kim et al. where delivery of 79 Cobalt Grey Equivalent (CGE) resulted in 90% tumour control rate at 5 years. However, a lower tumour dose of 62 CGE caused a precipitous drop in the control rate to only 50%, suggesting a steep dose–response relationship for local control.^38^ Although radiosurgical treatment approaches can deliver similar equivalent high doses, it does so with minimal or zero GTV to CTV margin and in a heterogeneous manner (‘hotspots’ within the target volume), and most series demonstrate recurrences that are outside the prescription isodose envelope.^39^

Contemporaneous proton therapy series show a local control rate ranging from 70 to 85% at 5 years, arguably better than that for comparable photon‐based treatment approaches. As local recurrences inevitably cause severe morbidity and eventually death, the improvement in local control is also reflected in better overall survival rates of 62–88.3% (Table 1). Reported series from the Paul Scherrer Institute, Switzerland, the pioneers of pencil‐beam scanning proton therapy, show excellent local control rates (5‐year LC = 75.8% and 7‐year LC = 70.9%) and long‐term survival (5‐year OS = 86.4% and 7‐year OS = 80%).^16^, ^40^ The authors also emphasised the need for chordomas to receive a higher dose compared to chondrosarcomas and another mesenchymal malignant tumour seen in the skull base region. This series being one of the largest long‐term outcomes of intensity‐modulated proton therapy (IMPT) for chordomas of the skull base showed reduced toxicity profiles in patients treated with proton therapy compared to historical cohorts.^16^ The largest reported experience of paediatric base of skull chordomas was reported from the Massachusetts General Hospital (MGH), Boston, by Ioakeim‐Ioannidou M et al.^41^ The group reported 10‐ and 20‐year PFS of 69% and 64%, respectively, with median survivals of 26 years and very low toxicity rates from high‐dose (median 76.7 Gy RBE) conventionally fractionated proton or combined proton–photon treatment approaches. It is of note that MGH transitioned from combined treatments to proton‐only treatments with the advent of pencil‐beam scanning technology. Several excellent reviews of the literature for proton therapy in skull base chordomas have been published recently including systematic reviews.^42^, ^43^, ^44^, ^45^, ^46^ Other significant series are summarised in Table 1.^15^, ^37^, ^47^, ^48^, ^49^

To date, there have been no randomised studies comparing proton therapy and photon therapy. In a meta‐analysis spanning the years 1999 to 2010, Di Maio et al.^50^ concluded that there was no significant difference in 5‐year overall survival based on the adjuvant radiation modality, whether it be proton therapy, carbon ion therapy, Gamma knife radiosurgery, or fractionated photon radiation. However, it is noteworthy that the impact of the radiation dose delivered was not explored. Therefore, it remains unclear whether the absence of modality‐related differences is attributable to the dose–response relationship. Interestingly, the analysis revealed a lower weighted mean 5‐year progression‐free survival (PFS) for patients treated with radiosurgical photon therapy when compared to carbon ion therapy (*P* = 0.042).

Carbon ion‐based particle therapy presents an alternative therapeutic approach for chordomas, characterised by a sharp distal fall‐off, with the added radiobiological benefit of higher radiobiological equivalent (RBE) doses of carbon ions. These RBE doses are generally considered to range from 1.5 to 6.7 at the distal edge.^51^ This may translate into further improving the therapeutic ratio by delivering a more biologically effective dose to this radio‐resistant tumour. While long‐term comparable data to PBT are missing, some encouraging early outcomes have been reported.^49^, ^52^ Currently, there is a single ongoing randomised controlled trial worldwide that compares the outcomes of skull base chordoma treatment using either proton or carbon ion radiation.^53^

### Reasons for recurrence and the value of proton therapy

Local recurrence is the most common site of progression for skull base chordomas. Depending on the length of follow‐up, recurrence rates for skull base chordoma vary between 40% and 60% at 10 years.^21^, ^54^ In cases where proton therapy is not available, clinicians may opt for stereotactic radiosurgery or fractionated stereotactic radiotherapy to extend the progression‐free interval and reduce local recurrence, sometimes in combination with a second‐look surgery. However, due to the necessity for exceptionally high radiation doses, as discussed earlier, this approach poses a substantial challenge in treatment planning. The clinical target volume, designed to encompass areas at risk of containing microscopic disease, often overlaps with critical neural and vascular structures that have been previously irradiated. Consequently, delivering such high radiation doses to adjacent normal tissues frequently leads to unacceptable levels of morbidity for the patient. Proton therapy, distinguished by its unique physical properties, offers a potential solution to both challenges. It enables dose escalation compared to conventional irradiation of the tumour while ensuring the safe delivery of doses to nearby sensitive structures. As demonstrated earlier, comparing patient series treated with radiosurgical approaches and proton therapy will always present challenges due to inherent selection biases, variations in treatment philosophy, and technical nuances. In the past, a radiosurgical approach may have been the only viable option when access to proton therapy was limited, primarily due to logistical and cost‐related barriers associated with travel. However, with the establishment of a proton therapy centre in Australia, making this advanced treatment accessible to Australians with chordoma, the approach to management warrants reconsideration. This development is expected to bring about a significant improvement in patient care, aligning it more closely with international best practices. This also affords a unique opportunity to generate prospectively collected, proton therapy‐related, long‐term toxicity data in this rare tumour, which may be the most robust data in the absence of randomised comparisons with other techniques. The ASPIRE^20^ aims to achieve this by establishing a survivorship plan for these patients and longitudinally following up in collaboration with the referring teams for delayed side effects of radiation, including visual, hearing, cognitive, endocrine, and growth‐related side effects as well as second malignancies, further comparing with a cohort of patients treated with photon‐based techniques.

There is uniform consensus among the global medical and patient community involved in the treatment of chordomas that local control and survival with proton therapy is superior when compared with conventional photon radiotherapy techniques.^55^ If using radiosurgical techniques, the concern for dose uniformity across the target volume is also highlighted in this paper.

A summary of recommendations is presented in Table 3.

**Table 3:** Recommended management of clival chordoma.

Aspect	Recommendation
Patient selection	All primary tumours should be selected for proton therapy where there is a dosimetric benefit to offer dose‐escalated radiotherapy to the majority of the residual tumour/tumour bed/ CTV OR for photon therapy where no such benefit is demonstrable. Recurrent tumours may be considered for carbon ion therapy under clinical trials, if available
Surgery	Maximal safe resection is the stated aim of surgery. Consideration must be given to the morbidity expected when more aggressive approaches are proposed – to be discussed with the patient and option of less aggressive surgery with adjuvant radiation to adequate doses may be preferable
Radiotherapy dose	At least 74 GyE EQD2 to be delivered to 95% of the high‐risk clinical target volume (CTV) at risk for residual disease. At least 54 GyE EQD2 to be delivered to 95% of the low‐risk CTV at risk for microscopic residual disease
Radiotherapy technique	At least IMRT/VMAT technique should be mandatory to deliver conventionally fractionated or hypofractionated dose schedules for photon‐based treatment. If using proton therapy passive‐scattered or pencil‐beam scanning modulated may be used
MDT	ALL CASES TO BE MANDATORILY DISCUSSED IN SKULL BASE MDT before primary treatment and at each recurrence
Volume, Expertise	Skull base MDT to include skull base surgeons with experience in chordoma surgery, radiation oncologist with experience/access to proton therapy, and skull base radiologist and medical oncologist

MDT, multidisciplinary team.

### The multidisciplinary team approach

The value of the multidisciplinary team approach in management of skull base chordoma is highlighted by Freeman et al., who published their experience of MD Anderson Cancer Centre, where they examined the outcomes of patients referred to their unit for initial management compared to patients referred after failed management at a different unit.^56^ They demonstrated that a comprehensive multidisciplinary team requires surgeons with expertise in complex open and endoscopic skull base surgery, radiotherapy teams that offer adjuvant therapy with protons and photons and have access to clinical trials for patients with progressive or metastatic disease to deliver superior patient outcomes.

Du et al. in 2022 published an article examining the influence of facility patient volume and outcome.^57^ The study demonstrated that academic centres operating on at least seven patients a year with skull base chordoma had superior outcomes compared to units who operated on fewer patients per year. It has also been demonstrated that delivery of radiotherapy in rare tumours like sarcomas, and thus arguably chordomas, is best done at specialised treatment centres.^58^ This an important concept to consider in Australia with a population of only 26.5 million people and an incidence of skull base chordoma of 0.033 per 100,000 population.^59^ Extrapolating this to the introduction of proton therapy to the region, there needs to be a robust system of multidisciplinary communication between geographically distant specialists and systematic referrals to a nationally accessible proton centre. This is because implementation of this technology is not without its challenges. It requires an experienced multidisciplinary team, rigorous patient selection criteria, and attention to detail in terms of surgical techniques and robust optimisation and evaluation protocols.

With the development of the first proton therapy centre in Australia, it is important to establish a collaborative approach and an associated multidisciplinary team of skull base surgeons, radiologists, pathologists, radiation oncologists, and medical oncologists, each with expertise in the various aspects of treating this rare and complex tumour, to ensure the best patient care will be delivered, as the evidence supports superior patient outcomes when complex cases are concentrated to a handful of expert multidisciplinary skull base teams. This may be enhanced by efforts to generate awareness about the specific requirements for proton therapy and establishment of a robust referral mechanism and access to clinical trials at the proton therapy centre.

## Conclusion

In Australia, the introduction of proton therapy will signify an important advancement in the treatment of clival chordomas, bringing the country in line with other world‐leading nations in the management of this rare tumour. This document emphasises the growing body of evidence in favour of proton therapy, underlining its potential to improve the management of clival chordomas in Australia. The forthcoming years will be instrumental in gauging the efficacy of the centre's deployment and its consequent influence on patient outcomes. It is imperative that continuous research and global collaboration be prioritised to guarantee the safe and proficient execution of this unprecedented Australian therapeutic approach.

## Conflict of Interest

The authors declare no conflict of interest.

## Funding Information

None.

## Data Availability

Data openly available in a public repository that issues datasets with DOIs.
